# Trends of Using Sensory Evaluation in New Product Development in the Food Industry in Countries That Belong to the EIT Regional Innovation Scheme

**DOI:** 10.3390/foods10020446

**Published:** 2021-02-18

**Authors:** Katarzyna Świąder, Magdalena Marczewska

**Affiliations:** 1 Department of Functional and Organic Food, Institute of Human Nutrition Sciences, Warsaw University of Life Sciences (SGGW–WULS), 159C Nowoursynowska Street, 02-776 Warsaw, Poland; 2 Department of Organization and Management Theory, Faculty of Management, University of Warsaw, 1/3 Szturmowa Street, 02-678 Warsaw, Poland; mmarczewska@wz.uw.edu.pl

**Keywords:** sensory evaluation, sensory analytical test, affective test, food industry, Regional Innovation Scheme (RIS), new product development (NPD)

## Abstract

Sensory evaluation plays an important role in New Product Development (NPD) in food industry. In the present study, the current trends of using sensory evaluation in NPD in the food industry in countries that belong to EIT Regional Innovation Scheme (RIS) were identified. The research was conducted in the first quarter of 2020. Computer assisted self-interviewing (CASI) technique for survey data collection was used. The sample included 122 respondents representing RIS countries that are the EU Member States and European Horizon 2020 Associated Countries that are classified as modest and moderate innovators according to European Innovation Scoreboard. The analysis presented in the paper allowed to describe the methods of sensory evaluation that can be used to support NPD in the food industry, identify the trends of using sensory evaluation in NPD in the food industry companies in RIS countries. The research results showed that almost 70% of companies apply sensory evaluation methods in NPD. The larger the company, the more often the methods of sensory evaluation are used in NPDs. Almost 60% of companies employing 51–100, 101–1000 and more than 5000 people, respectively declare the use of expert (analytical) test. However, regardless of size, most companies prefer consumer (affective) test to expert tests. Based on the results, it seems that the potential of usage sensory evaluation methods is not yet fully exploited in the food industry.

## 1. Introduction and Background

One of the basic conditions for the development of the company and its long-term success is its innovation [1,2,3]. Innovative companies are able to respond to the challenges of the world around much faster and more effectively than non-innovative ones [1,4]. Companies, both small and medium enterprises (SMEs) and large corporations, have begun to consider innovation as an integral part of their strategy in order to create a lasting competitive advantage and to adapt the products or services offered to the needs of consumers, which has led to a greater need for teams mainly involved in the development of new products [1].

New product development (NPD) is “the process of designing a new product, producing it and bringing it to market” [5]. The definitions of the new product proposed by Fuller [6] are as follows “A product not previously manufactured by a company and introduced by that company into its marketplace or into a new marketplace” or “The presentation or rebranding by a company of an established product in a new form, a new package or under a new label into a market not previously explored by that company”. NPD is based on customized procedures, models and is supported by appropriate tools; thus, it brings significant benefits in terms of production costs, product quality and supply chain availability, which is crucial for success and business development [5]. Moreover, it is one of the ways to increase the company’s profitability [6]. However, we must bear in mind that the success of a new product depends on many factors and in some cases the development of a new product may involve a high risk [5,7,8]. 

There are many different types of new food products on the market, such as line extensions [6,8] (e.g., new flavors for an ice-cream), repositioned existing product [6] (e.g., oil as one of the main constituents of mayonnaise or vegetable paste), new form or size of existing product [6] (e.g., instant oatmeal in ready to eat cup), reformulation of existing product [6] (e.g., sugar-reduced or sugar-free cakes), repackaging of existing product [6] (e.g., infant food containers changed from glass to squeezed), innovative products [6] (e.g., the plant-based analogue of tuna meat), creative products [6] (e.g., 3D printing food). However, one must remember that consumers purchase products regardless of their category, but rather because they meet their needs [9]. The growing awareness of consumers and their needs for natural, safe and healthy food [10] as well as consumers preferences for personalized, customized food products have led to diversity in the food market [5]. These changes led to new food trends such as functional food [10,11] with their healthful properties and nutritional value [10,12], novel food or use of nanotechnology in food sector [11]. 

It is not difficult to develop a recipe for a new product, but it is difficult to develop a new product that meets the expectations of the assumed number of consumers and is profitable to sell [8]. Every manufacturer’s dream is to have their new product on the shelf as soon as possible and to sell it with great success, but the pathway from idea to shelf can be long and winding and leads through different stages of the process of designing new products. New product process proposed by Cooper [13] assumes the following stages: (1) new product idea; (2) costumer defined, internally defined, idea refined; (3) project formally opened; (4) development; (5) prototyping; (6) field trials; (7) launch. O’Sullivan [8] presents NPD process from the inception to the shelf divided into the following steps: (1) ideation; (2) project pre-planning; (3) validation of proof of concept; (4) process optimization and up-scaling; (5) commercialization; (6) pre and post-approval and shelf-life testing. The commonly used framework listing the main stages in the NPD process is based on four stages: (1) opportunity identification; (2) product design and development; (3) testing; (4) introduction and launch [14]. Azanedo [5] points out, however, that currently in the development of new food products (NFPD), the NPD models used need to be modified in order to better and more effectively support the food industry. Moreover, the study suggests that the most attention should be paid to areas such as meeting consumer demands, consumer preferences and sensory characteristics of products, taking into account the seasonality of ingredients and the traceability and safety of final products, and the large-scale production of food with respect for the environment and the supply and distribution of local ingredients. 

In the process of designing food products, it is important to recognize the needs of the customer, direct the designed product towards them, and then communicate and explain the value of this designed product to the consumer [9]. Consumers buy image, comfort, nutrition, using their senses, sensory sensitivity, they buy sensory properties. That is why sensory methods are an important, integral tool that should be used in NPD process. When designing products, the most important quality feature of a product is its direct relationship to satisfaction, perception and ultimate acceptance by the consumer of the sensory qualities of the product [8,15].

Sensory evaluation and new product development are strongly linked. Sensory analysis methods can be used at many stages of the design process to assess the quality of the product and the expectations of consumers and their reactions to the product. Following the framework indicating the importance of sensory evaluation in NPD [5,8,15,16,17,18,19,20,21] this empirical research aims to identify the trends of using sensory evaluation in New Product Development in the food industry companies in countries that belong to EIT Regional Innovation Scheme (RIS) by addressing research questions listed below. There are only a few studies tackling application of sensory evaluation methods in food industry companies that focus on general trends and compare the use of sensory evaluation methods between companies of different size and those that value the most diverse stages of the NPD process. Thus, this paper seeks to fill this research gap by addressing the following research questions:

RQ1 What methods of sensory evaluation can be used to support NPD in the food industry?

RQ2 What are the trends of using sensory evaluation in NPD in the food industry companies in countries that belong to EIT Regional Innovation Scheme (RIS)?

RQ3 What sensory evaluation methods are used by companies that tend to value the most a specific stage of the NPD process? 

RQ4 What are the differences in applying sensory evaluation methods among companies of different sizes from RIS countries?

The first research question will be answered based on desk research results and literature review, whereas the answers to the questions 2-4 will be based on the results of empirical research.

The article proceeds as follows. First the research background along with the research aims are presented in the introductory part. The next section of the paper is devoted to sensory evaluation methods, their use and importance in NPD process with the focus on food industry. The third part of the paper describes methods used for the analysis, along with data selection and extraction process. It is followed by the presentation of results of a pilot study aimed to identify the trends of using sensory evaluation in New Product Development in the food industry in countries that belong to EIT Regional Innovation Scheme (RIS) and its discussion. The paper ends with concluding remarks and future research directions. 

## 2. Sensory Evaluation in New Product Development

Sensory tests have been used since people began to assess everything that can be used by them and to distinguish between good and bad, from water and food, starting with and ending with weapons and other objects. The increase in trade, on the other hand, has inspired the formal application of sensory testing significantly [17].

The history of “sensory” analysis also dates back to the wars, when efforts were made to provide the American forces with acceptable food [17]. The early 1900s gave rise to a professional taster and consultant in emerging industries food, beverages, and cosmetics. The term “organoleptic examination” was then used to describe allegedly objective sensory characteristics. However, these tests were still often subjective rather than objective [17].

International interest in food and agriculture in the mid-1960s and on into the 1970s, the energy crisis, food production and raw material costs, competition and market internationalization have created opportunities for sensory evaluation [22]. The course of events has made sensory evaluation a recognized scientific specialty [17,22]. Sensory evaluation is defined as “a scientific discipline used to evoke, measure, analyze and interpret reactions to those characteristics of foods and materials as they are perceived by the senses of sight, smell, taste, touch and hearing” [18,22,23]. Sensory evaluation, like other scientific methods in which we take measurements, is based on taking measurements in a precise and accurate way, considering the sensitivity and aiming at avoiding false-positive results [24]. In order to be considered a reliable method, sensory analysis must be based on the skills of a sensory analyst to optimize definition of the problem (what should be measured) and test design (produce the desired accuracy of results), instrumentation (selected and trained panelists) and interpretation of results [17,18].

When assessing the characteristics of a food product, we first assess its appearance, then its odor, texture/consistency and flavor/taste [17]. The reaction to a sensory stimulus, on the other hand, can be divided into three different dimensions: qualitative perception, quantitative perception, and hedonic reaction [25]. In order to obtain that information, we must use analytical or affective methods during the sensory evaluation [16,17,22,25]. 

The purpose of analytical tests is to assess in detail the sensory quality of a product, while affective tests are used to measure the acceptability or preference of a product by consumers [8,25]. The basic goal while choosing sensory evaluation methods is to match the right test with the right question that we want to answer. Among the analytical tests (Table 1) that are mainly evaluated by the panel experts, we can use the discrimination test to determine if there are sensory differences or similarities between products, without describing their nature. We can use the Triangle test, Duo-trio test, Two out of five test. As far as the nature of the differences between products is known, we can use a grading test such as paired comparison test, to position different products according to their sensory characteristics. A ranking test can be used to assess noticeable differences between several products depending on the intensity of the difference, and a scoring test may be used to assess the specific intensity of the sensory characteristics of products. In the analytical test, a descriptive test (called sensory profiling) is very often used to describe and evaluate both the intensity and quality of perceived product characteristics, i.e., Quantitative Descriptive Analysis®, Texture Profile® [8,18,22,25,26].

Apart of above-mentioned methods, there are new one called rapid sensory evaluation methods, that are more flexible, simple and easy to perform and can be used with semi trained assessors or naive assessors such as: flash profiling, ultraflash profiling, ranking test, napping, free sorting, optimized descriptive profiling, ideal profile method, check-all-that-apply, temporal dominance of sensation [8].

Sensory acceptance of the product by the consumer, its hedonic reaction, can be assessed using an affective test (Table 1). This may be a paired comparison test, in which the consumer chooses the sample he or she prefers or likes most from two or more, or a ranking test, in which the consumer rank the product according to his or her preferences, whereas in order to determine the scale of preference among the products or the degree of pleasure/liking the product gives, a hedonic scoring test with scales can be used [22,25,27]. An example of a qualitative affective sensory test is the focus group, a rapid method to test the product and packaging concepts and ideas [8]. 

Sensory evaluation of a product, including both the analytical sensory evaluation carried out by a panel of experts and the affective test carried out on consumers, allows to obtain more information about the product being analyzed, its quality and to verify factors influencing its acceptability by consumers, which facilitates work on improving the quality of the product or its reformulation [10].

It is quite common practice in food companies to use inappropriate sensory analysis methods for specific research purposes [8].

Both affective tests and analytical (expert) sensory tests can be use on each step of new product development (Table 2). During ideation, the initial project planning and validation of proof-of-concept affective test, such as focus groups, can be used, but also methods such as free elicitation, information acceleration (IA), Kelly repertory grid, laddering, lead user technique and Zaltman metaphor elicitation technique (ZMET) are recommended. The stage where both the affective test and the sensory test can be applied is process optimization and up-scaling, where the sensory acceptance test (affective test) can be applied, as well as the analytical test: a descriptive test such as the Quantitative Descriptive Analysis® and a rapid test such as the Ranking Descriptive Analysis. A very important aspect during the commercialization of a product is to carry out sensory Acceptance Tests. Carrying out Consumer Tests is also very important during pre- and post-approval tests and product durability tests [8]. 

Besides new product development, sensory evaluation can also be used in other product development activities, such as product prototype evaluation [8,28]; product concept fit [8,28]; pilot plant scale-up; cost reduction study by substituting or modifying ingredients [28]; process change [29,30]; ingredients changes for example caused by reduction of salt [20,21,31], sugar [21,32,33], or fat [21,31] or purchase specifications change [19] as well as product improvement and optimization of product formula [34,35].

Moreover, sensory evaluation methods are used to supports marketing and marketing research activities [19,28], beginning with new product development and assessment of market potential, continuing through tracking product performance, and contributing to special assignments such as developing tests. They can be used in sensory marketing as data to support or challenge advertising claims [36]. Sensory marketing defined as “ marketing that engages the senses of consumers and influences their perception, judgement and behavior” examines how acoustic, tactile and olfactory sensory stimuli influence decision making processes and the formation of consumer attitudes and can thus be used for advertising design and effectiveness [36].

Sensory evaluation is also used to compare the quality of competing products [37]. Consumer test can be used to define the most important characteristics of food affecting purchasing decisions, identify preferences and to know consumers when purchasing food products [12,37].

Sensory evaluation methods can be used in several food industry departments; however, they are mainly used for quality control and product research and development (R&D) in big companies [17,19] so their potential is not yet fully exploited in the food industry.

Sensory evaluation methods can be used for shelf-life assessment of food products [38,39,40] and new technologic that can extend product durability and quality such as pulsed electric fields [29,30]. Changes in the sensory characteristics of food products affect the determination of their shelf-life, and the freshness of a product’s safety and quality are characteristics to which consumers are now paying increasing attention [40]. 

Expert tests such as sensory descriptive analysis and consumer test can be well used to investigate exotic, authentic, ethnic, or artisanal foods [23] such as green tea [41,42], soy sauce [43,44], kimchi [45,46], tofu [47,48], dates [49,50] as well as innovative one such as tea-infused yoghurts [10] or plant-based yogurts made from almond, cashew, coconut, hemp or soy [51].

There are many possible ways to apply sensory evaluation in NPD in the food industry. Companies can use analytical and affective (hedonic) sensory tests and choose from a variety of sensory methods that can be used at different stages of the NPD process and allow the different characteristics of food products to be studied and consumer reactions to these products and their expectations.

## 3. Materials and Methods

The study was conducted as a part of the project “Summer school on the New Product Development for the food industry” (2020 edition) financed by the EIT Food under Horizon2020, which aimed to address contemporary challenges related to NPD in the food industry. The research was carried out as a pilot study (part of the project) to identify the trends of using sensory evaluation in New Product Development in the food industry in countries that belong to EIT Regional Innovation Scheme (RIS); thus, the research sample was composed of respondents representing these countries. The EIT’s Innovation Communities are strengthening the innovation ecosystem in parts of Central, Eastern and Southern Europe and have been set up to increase the number and business maturity of start-ups coming from these regions [52].

The EIT RIS countries are the EU Member States and European Horizon 2020 Associated Countries who are classified as modest and moderate innovators according to European Innovation Scoreboard [53]. These are Bulgaria, Croatia, Cyprus, Czech Republic, Estonia, Greece, Hungary, Italy, Latvia, Lithuania, Malta, Poland, Portugal, Romania, Slovakia, Slovenia and Spain, as well as Albania, Armenia, Bosnia and Herzegovina, Faroe Islands, Georgia, Moldova, Montenegro, Republic of North Macedonia, Serbia, Turkey and Ukraine. Modest and moderate innovators show an innovation performance below the EU average. Since NPD is one of the dimensions of countries’ innovation performance, it seems important to analyze it, especially in the context of countries which are not best performers, in order to identify the ways to boost its development, as well as explore and design new potential pathways to support it. 

The data used in this research were collected through computer assisted self-interviewing (CASI). This technique allows to collect data from respondents who complete the survey questionnaire via computer without any external assistance. The use of such research method is based on the assumption that respondents can read and understand the questions well enough to give precise answers [54]. The questionnaires were conducted in the first quarter of 2020, before the CODIV-19 pandemic. 

The sample included 122 respondents representing RIS countries. All participants have agreed to participate in the study and received a link to the questionnaire for them only. If respondents provided their questionnaires with missing/unreliable data, they were excluded from the sample analyzed (*n* = 8).

The respondents were employed in companies from the food industry and performed various jobs related to NPD. Most of them were food technologists, project/program managers or C-level executives. The characteristics of the respondents are presented in (Table 3). Respondents differ in gender, age and length of professional experience in the food sector. 

The respondents were employees of food industry companies of various sizes, where SMEs constituted almost three quarters of the sample. Further, almost 40% of the sample were micro enterprises with fewer than 10 employees and around 20% represented small enterprises. Table 4 presents details on sample characteristics. 

The questionnaire was composed of questions of different types, i.e., single-choice questions and open-ended questions. Most of the questions had already defined answers to choose from; however, in many cases, there was space left for participants to write additional comments. The questionnaire used in the study was related to various aspects of NPD and sensory analysis, including average length of NPD project, most important stages of NPD, application of sensory evaluation in NPD, sensory methods used in NPD, use of consumer tests to verify consumer preferences or acceptance for the developed product and use of expert tests performed by a panel of trained experts to determine the quality of products. The obtained data were analyzed considering the sample of companies from RIS countries in general, and also taking into account the specificity of companies of different sizes and comparing the importance of a specific stage of NPD and its impact on the use of sensory evaluation. Sample survey questions and answers are presented in (Table 5).

## 4. Results and Discussion

On the basis of the studies carried out, it is worth pointing out that the NPD projects in the analyzed food sector companies are relatively short (Table 6), i.e., 34.4% of the companies claim that they manage to develop and introduce an entire project within a year, and 41.8% need less than 2 years to complete it. The companies argue that due to market dynamics and growth they have to act fast in order to be competitive.

One of the main factors influencing product development is the speed with which the product is placed on the market. If this process took too long, the research previously carried out may no longer correspond to reality, e.g., demographic consumer segments initially identified as potential buyers and optimistic users may have changed their minds about wanting to buy the product [8]. Moreover, because the development of a new product may involve a high risk [5,7,8] it is worth running the NPD process relatively fast.

According to Dijksterhuis [55], 50 to 75% of newly developed products placed on the market are disposed of, which is far from the assumed financial targets. More than 90% (some say it is even 98%) of all NPDs in the food and drink industry fail, while the remaining 10% (or rather 2%) have been extremely successful, and the final prize is huge [8]. The main research problem behind the high failure rate of new products is the lack of understanding of consumers’ motivation and choice. Therefore, the research on consumer behavior should be used more effectively to address this problem [55].

Sensory evaluation seems to be an important element of NPD in RIS countries. 67.2% of analyzed companies claim to apply sensory evaluation methods while working on new products.

Sensory food science is a discipline that is increasingly used and needed in order to better understand the factors influencing consumer preferences. Sensory evaluation is an essential tool for use by the food industry now and in the future, when, due to social and industrial needs that are consumer-oriented, their use will increase in the future [18].

There are differences in applying sensory evaluation among companies from RIS countries, which value the most different stages of NPD. It is not surprising that the use of consumer assessment tests, expert tests and other sensory evaluation methods is most common among companies, which see “sensory quality of the product and its acceptability by consumers” as the most important stage of NPD process (Table 7). However, “developing a product recipe, selection and safety of raw materials, its health-promoting properties” is seen as crucial in the NPD process by more than one fourth of the researched companies and within this group sensory evaluation, along with consumer assessment and expert tests are also seen as valuable. Table 7 presents the use of various sensory evaluation methods by companies that tend to value the most a specific stage of the NPD process. E.g., 74.4% of companies that see as the most important stage of NPD “developing a product recipe, selection and safety of raw materials, its health-promoting properties” apply sensory evaluation in NPD, 66.7% use consumer tests, and 46.2% use expert tests. This means that there are some companies that use both types of tests, and other focus only on one specific test.

In theory, sensory analysis is applied at many stages of new product development. Affective tests, e.g., focus group, can be used at the product concept stage, while from the assessment of a prototype, affective methods such as examining product preference or acceptability can be used. Analytical research, e.g., discrimination tests, can be used to optimize the product, e.g., nutritional optimization of the product related to the reduction or exchange of sucrose in the product. However, to determine the quality of the product prototype and the differences between the variants, it is worth using Quantitative Descriptive Analysis (QDA®). In order to obtain more complete information about the product, descriptive analysis can be carried out in parallel with sensory affective analysis and consumer testing and instrumental measurements. Before a product is placed on the market, Multivariate Data Analysis (MVA) can be used to correlate sensory descriptive analysis with factors affecting the consumer [8].

**Figure 1:**
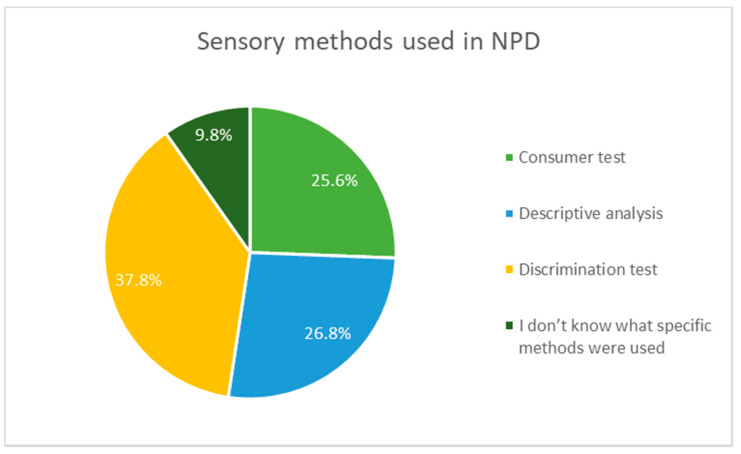
Sensory methods used in NPD.

There are three most popular sensory methods used in NPD in the food sector in RIS countries (Figure 1), and discrimination test is the most popular one (37.8%). However, companies usually use more than one method in order to verify, compare and combine the results of sensory methods used.

The discriminatory test is a powerful sensory evaluation method in terms of its sensitivity, which provides reliable and important results that, because of its effectiveness, have saved companies a considerable amount of time, money and effort [22].

Sensory methods can be used at many stages of the NPD, so it is important to use them at many stages, but you can see that discriminatory methods are most often used, and the use of methods depends on the purpose of the research.

**Figure 2:**
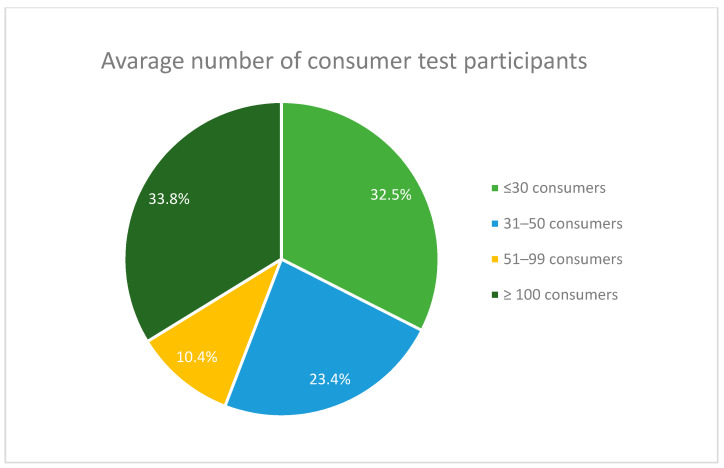
Average number of consumer test participants.

Companies from the sample tend to value consumers opinions. Of the companies, 63.1% use consumer tests (affective tests) to verify consumer acceptance or preferences for the developed product. Interestingly, the average number of consumer test participants differs significantly between the companies form the sample. On the one hand, almost 33% of companies collect information from a small number of consumers, i.e., ≤30. On the other hand, over 33% conduct consumer assessment on relatively big samples of more than 100 consumers (Figure 2). As confirmed by other researchers, consumer acceptance testing can be used during the product development and optimization based on 25–75 individuals, while a large number of consumers (more than 100) is used in consumer tests before product lunch [8].

Interest in consumer (hedonic) research in both basic psychophysics and applied and consumer food research has increased significantly in recent years. Research on the identification of differences in the hedonic response to chemical stimuli has become the basis for a better understanding of the role of sensory, perceptual, cognitive and genetic factors influencing consumer food preferences and choice. Now that the consumer market has become more crowded and competitive, applied product research is not only investigating which products are more popular with consumers than others, but it has become more important than ever to discover the basic segmentation of consumers [56].

Compared to other types of sensory evaluation methods, expert tests performed by a panel of trained experts to determine the quality of products seem to be rather unpopular among companies representing food industry companies from RIS countries. Only 42.6% of these companies perform expert tests. There are several primary reasons mentioned by the food industry companies from RIS countries why such tests are not popular. First, these companies do not employ such experts and are not willing to outsource such service. Second, these companies have no knowledge or experience in this field, thus prefering to use other, better known methods of sensory evaluation. Third, the sensory quality of products is assessed by company owners, management board or employees and friends. Among secondary reasons for not using such tests, companies list high expenses and lack of need. However, looking at the answers given by the companies from the sample, along with additional justifications, it seems that companies representing the food industry in RIS countries do not have sufficient knowledge in the field to use expert tests.

The use of sensory evaluation methods differs among companies of various sizes from RIS countries. In general, the bigger the company, the more popular and used sensory evaluation methods in NPD (Table 8). Most of the companies, regardless of size, prefer consumer assessment tests over expert tests. However, almost 60% of companies employing 51–100, 101–1000 and above 5000, respectively, declare to use expert tests. These results seem to confirm the research results presented by other authors stating that the bigger the company, the wider knowledge and application of sensory evaluation methods. 

Consumer assessment tests run by companies of different sizes vary in average number of consumer test participants. Almost 60% of companies employing 1–10 employees run these tests on the sample of ≤30 consumers. Companies employing 11–50 and 51–100 employees prefer samples of 31–50 or more than 100 consumers; more than 40% of companies employing 101–1000 people conduct these tests with more than 100 consumers, whereas 33.3% of those employing 1001–5000 run tests on the samples of ≤30 and more than 100 consumers, respectively. Not surprisingly, almost 82% of the biggest companies tend to research more than 100 consumers at a time.

When it comes to specific sensory methods used in NPD by companies of different sizes, consumer acceptance tests are the most popular among those employing 1–10 and 1001–5000 employees, whereas discrimination tests are more widely used by those employing 11–50, 101–1000 and above 5000 people.

Sensory evaluation is beginning to be applied in many food companies, and research results of other authors also confirm that its adoption depends, among others, on the size of the company [19]. For example, in the case of large companies such as Puleva Biotech S.A., Spain, sensory testing is carried out daily in several departments, i.e., quality control, research and development and marketing. In the case of small companies, the situation is completely different. Small companies do not have the structure, personnel and/or qualifications to carry out sensory research, although they are aware of its existence and effectiveness. Medium-sized companies try to include sensory evaluation as one of the modern tools to improve their efficiency and thus their income [19].

All in all, sensory food research can contribute to understanding consumer response to emerging trends in food production, processing and consumption. In order to make better use of sensory research, it is necessary to allow access to appropriate university training programmes, funding for fundamental research and multidisciplinary cooperation [18].

## 5. Conclusions

The analysis presented above, based on desk research and CASI, allowed to answer the research questions (RQ) outlined in the introductory part of the paper. 

Thereby RQ1 allowed to describe the methods of sensory evaluation that can be used to support NPD in the food industry. Companies can benefit from the many achievements of the scientific discipline of sensory evaluation, and they can use analytical and affective sensory tests. They have many different sensory methods at their disposal, which can be used at different stages of the NPD process, from the idea and conception of the product to its launch and subsequent approval, which allows them to assess both the quality of the product and the factors influencing consumers and their purchasing decisions.

RQ2 addressed the trends of using sensory evaluation in NPD in the food industry companies in RIS countries. The research results showed that almost 70% of companies apply sensory evaluation methods in NPD; however, among them there are the following three most popular ones: discrimination test, descriptive analysis and consumer test. Moreover, the companies generally value consumers opinions and more than 63% of them uses consumer assessment tests to verify the match between the products they offer and expectations of consumers. Nevertheless, looking at the answers given by the companies from the sample, along with their additional justifications, it seems that companies representing food industry in RIS countries do not have sufficient knowledge in the field to use expert tests, which may lead to the use of unsuitable methods of sensory analysis to achieve the research objectives set companies.

RQ3 was aimed at identifying sensory evaluation methods that are used by companies, which mostly tend to value a specific stage of the NPD process. First, it allowed to identify that more than one fourth of the companies from the sample see “developing a product recipe, selection and safety of raw materials, its health-promoting properties” as the most important stage of the NPD process, almost one fifth values the most “creating a new product, idea”, whereas around 10%, respectively, treat “ensuring the safety of produced food” and “sensory quality of the product and its acceptability by consumers” as highly important. Second, the analysis allowed to link specific sensory methods used with companies valuing different staged of NPD. Almost 82% of companies that value the most “sensory quality of the product and its acceptability by consumers” apply sensory evaluation methods in NPD; 73% use consumer assessment tests, and 64% use expert tests. The knowledge of sensory evaluation methods and their application is really high in this group of companies, compared to the whole sample. Among other groups of companies identified based on the stage of NPD process they value, 70% of companies focused on “creating a new product, idea” use consumer assessment tests and 67% of those focused on “developing a product recipe, selection and safety of raw materials, its health-promoting properties”. The knowledge and application of different sensory evaluation methods is diverse among companies representing different groups; however, interestingly, companies focused on “obtaining funds/grants” and “product distribution” seem to disregard expert tests.

RQ4 allowed to characterize the differences in applying sensory evaluation methods among companies of different sizes from RIS countries. Sensory evaluation is increasingly being used in many food companies, and the use of these methods depends to a large extent on the size of the company. Among the companies analyzed in the RIS countries, it can be seen that the larger the company, the more often the methods of sensory evaluation are used in NPDs. Almost 60% of companies employing 51–100, 101–1000 and more than 5000 people, respectively, declare the use of expert (analytical) test. However, regardless of size, most companies prefer consumer (affective) test to expert tests. Consumer tests are most popular among companies with 1–10 and 1001–5000 employees, while discrimination tests included in analytical test are more frequently used by companies with 11–50, 101–1000 and over 5000 employees.

All in all, it seems that the potential of usage sensory evaluation methods is not yet fully exploited in the food industry. Since this pilot study has been carried out on a sample including many countries from a specific group (RIS countries), a further study is planned to analyze a specific group of companies from selected RIS countries in order to take a closer look at specific sensory analysis methods used in the development of selected food products.

Drawing on research results presented above, future research directions in investigating the importance of sensory evaluation in NPD in the food industry may also include the following topics: focus on analytical test or affective test, one sensory method, one method and one country only; focus on SMEs from specific branch of food industry in RIS countries and/or comparison with other countries; focus on size and experience of food industry in RIS countries; focus on specific stage of NPD and the use of sensory evaluation.

## Figures and Tables

**Table 1:** Types of most popular analytical and affective tests used in sensory evaluation.

	Type of Test	Question
ANALYTICAL TEST	Discrimination test: Triangle Duo-trio Two out of five	Which sample is different? Which sample is different from the reference sample? Which 3 samples are the same type?
	Grading test: Paired-comparison test Ranking test Scoring test	Which sample is most (sweet, bitter, etc.)? List the samples in increasing order of intensity for a selected attribute (sweet, bitter, etc.)? How (sweet, bitter, etc.) the sample is?
	Descriptive test: Quantitative Descriptive Analysis® Flavor Profile® Texture Profile® Spectrum^TM^ Descriptive Analysis Free-Choice Profiling	Are the products different and how do they differ?
AFFECTIVE TEST	Paired comparison test	Which do you prefer? Which do you like most?
	Ranking test	Rank this product by preference?
	Hedonic scoring test	Asses the degree of pleasure/liking given by products on the scale?

Source: Own elaboration based on [8,18,22,25,26,27].

**Table 2:** Use of sensory evaluation methods on each step of new product development.

Stages of New Product Development	Applied Sensory Evaluation Methods	
	Affective Test	Analytical Test
Ideation	Focus Groups, Free Elicitation, IA *, Kelly Repertory Grid, Laddering, Lead User Technique, ZMET *	-
Project Pre-Planning		
Validation of Proof of Concept		
Process optimization and up-scaling	Sensory Acceptance Testing	Descriptive test: QDA^®^*
		Rapid test: RDA *
Commercialization	Sensory Acceptance Testing	-
Pre- and Post-Approval and shelf-life testing	Consumer Testing (*n* > 100)	-

IA *, information acceleration; ZMET, Zaltman metaphor elicitation technique; QDA^®^, Quantitative Descriptive Analysis; RDA, Ranking Descriptive Analysis. Source: Own elaboration based on [8].

**Table 3:** The characteristics of the respondents (*n* = 122).

Characteristic		*n* *	%
Gender	Male	73	59.8%
	Female	49	40.2%
Age group	18–24	20	16.4%
	25–34	77	63.1%
	35–44	22	18.0%
	45–54	2	1.6%
	55 or more	1	0.8%
Professional experience in food sector	less than 1 year	36	29.5%
	between 1 and 2 years	27	22.1%
	between 2 and 5 years	31	25.4%
	between 5 and 10 years	16	13.1%
	more than 10 years	12	9.8%
Area of expertise	Food science/chemistry/technology	23	18.9%
	Food safety/quality	16	13.1%
	Product development in the food sector	15	12.3%
	Food production/manufacturing/processing	13	10.7%
	Entrepreneurship/business startup/development/ acceleration in agri-food or life sciences	11	9.0%
	Marketing/consumer behavior/market research, preferably in the food sector	10	8.2%
	Nutrition/food related health	8	6.6%
	Other	7	5.7%
	Agriculture/agricultural technologies	6	4.9%
	Consumer testing/sensory science	4	3.3%
	Food-health nexus	3	2.5%
	Food waste/side stream valuation	2	1.6%
	New business models	2	1.6%
	Bioeconomy/resource stewardship/sustainability	1	0.8%
	** STEM/STEAM/science education	1	0.8%

* number of respondents. ** STEM, Science, Technology, Engineering and Math; STEAM, Science, Technology, Engineering, Arts and Math.

**Table 4:** Sample characteristics (*n* = 122).

Characteristic		*n* *	%
Number of employees in organization	1–10	48	39.3%
	11–50	23	18.9%
	51–100	12	9.8%
	101–1000	21	17.2%
	1001–5000	6	4.9%
	Above 5000	12	9.8%
Country **	ES	19	15.6%
	IT	17	13.9%
	GR	14	11.5%
	PL	14	11.5%
	HU	8	6.6%
	TR	7	5.7%
	PT	6	4.9%
	BG	4	3.3%
	HR	4	3.3%
	CZ	3	2.5%
	EE	3	2.5%
	LT	3	2.5%
	ME	3	2.5%
	RO	3	2.5%
	RS	3	2.5%
	AL	2	1.6%
	SI	2	1.6%
	LV	2	1.6%
	SK	2	1.6%
	UA	2	1.6%
	GE	1	0.8%

** AL, Albania; BG, Bulgaria; CZ, Czech Republic; EE, Estonia; ES, Spain; GE, Georgia; GR, Greece; HR, Croatia; HU, Hungary; IT, Italy; LT, Lithuania; LV, Latvia; ME, Montenegro; PL, Poland; PT, Portugal; RO, Romania; RS, Serbia; SI, Slovenia; SK, Slovakia; TR, Turkey; UA, Ukraine. * Number of respondents.

**Table 5:** Sample survey questions and answers.

General Questions	
Question	Answer
Country	(open ended question)
Gender	Male Female
Age group	18–24 25–34 35–44 45–54 55 or more
Please indicate your area of expertise	Agriculture/agricultural technologies; Bioeconomy/resource stewardship/sustainability; Consumer testing/sensory science; Entrepreneurship/business start-up/development/acceleration in agri-food or life sciences; Education/andragogy, in particular in entrepreneurship or food systems; Food-health nexus; Food production/manufacturing/processing; Food science/chemistry/technology; Food safety/quality; Food systems/food value chains; Food waste/side stream valuation; Marketing/consumer behavior/market research, preferably in the food sector; New business models; Nutrition/food related health; Product development in the food sector; Science communication/public engagement of science/citizen science; * STEM/STEAM/science education; Trust/transparency; Other
How long is your professional experience in the food sector?	less than 1 year; between 1 and 2 years; between 2 and 5 years; between 5 and 10 years; more than 10 years
How many employees are there in your entire organization?	1–10 11–50 51–100 101–1000 1001-5000 Above 5000
Which role best describes your current position in the company?	C-Level Executive (*CEO, CTO, etc.) Development Leadership: VP/Director Development Direct Manager: Team leader/Group Leader Development Team Member: Architect/Developer/*QA Project/Program Manager System Engineer Product Manager/Product Owner DevOps Engineer External Consultant/Trainer Food technologist Sensory and Consumer Manager Other (please specify below)
Industry specific questions	
Question	**Answer**
According to the best of your knowledge, what is the average length of NPD project in your organization? Please take into account the time from ideation to commercialization.	Less than 1 year 1–2 years 2–4 years More than 4 years
Which stages of food design are the most important in the company you work for? Please select all that apply.	a. Creating a new product, idea b. Developing a product recipe, selection and safety of raw materials, its health-promoting properties c. Packaging, its appearance, functionality, impact on product durability and the environment d. Labeling in accordance with legal requirements e. Ensuring the safety of produced food f. Sensory quality of the product and its acceptability by consumers g. Product distribution h. Product marketing and advertising i. Obtaining funds/grants j. Other (please specify)
Does the company you work for apply a sensory evaluation test in the product development?	Yes/No
What kind of sensory methods are used by the company you work for in the process of food product design? Please select all that apply.	a. Discrimination test b. Descriptive analysis c. Consumer test d. Other (please specify) e. I don’t know what specific methods were used
Does the company you work for perform consumer tests to verify consumer preferences/acceptances for the developed product?	Yes/No
What is the average number of consumer test participants that the company you work for performs?	a. ≤30 consumers b. 31–50 consumers c. 51–99 consumers d. ≥100 consumers
Does the company you work for use expert tests performed by a panel of trained experts to determine the quality of products?	Yes/No
If company you work for does not use expert tests performed by a panel of trained experts to determine the quality of products, please indicate why not. Please select all that apply.	a. The company does not employ such experts b. Such research is too expensive c. Such research is not necessary when designing products d. The sensory quality of products is assessed by company owners, management board or employees and friends e. The company has no knowledge or experience in this field f. Other (please specify)

* STEM, Science, Technology, Engineering and Math; STEAM, Science, Technology, Engineering, Arts and Math; CEO, chief executive officer, CTO, chief technical officer, QA, Quality Assurance.

**Table 6:** Average length of New Product Development (NPD) project.

Average Length of NPD Project	*n* *	%
Less than 1 year	42	34.4%
1–2 years	51	41.8%
2–4 years	22	18.0%
More than 4 years	7	5.7%

* Number of respondents.

**Table 7:** Most important stages of NPD and use of sensory evaluation.

	% of Companies Indicating Selected Stage as the Most Important NPD Stage	Application of Sensory Evaluation in NPD	Use of Consumer Tests	Use of Expert Tests
Developing a product recipe, selection and safety of raw materials, its health-promoting properties	32.0%	74.4%	66.7%	46.2%
Creating a new product, idea	24.6%	66.7%	70.0%	40.0%
Ensuring the safety of produced food	13.9%	70.6%	52.9%	41.2%
Sensory quality of the product and its acceptability by consumers	9.0%	81.8%	72.7%	63.6%
Packaging, its appearance, functionality, impact on product durability and the environment	5.7%	71.4%	57.1%	42.9%
Product marketing and advertising	5.7%	57.1%	57.1%	42.9%
Obtaining funds/grants	2.5%	33.3%	66.7%	0.0%
Product distribution	2.5%	0.0%	33.3%	0.0%
Labeling in accordance with legal requirements	0.8%	100.0%	100.0%	100.0%
Other	3.3%	25.0%	25.0%	25.0%

**Table 8:** Use of sensory evaluation in companies from RIS countries by company size.

	Application of Sensory Evaluation in NPD		Use of Consumer Assessment Tests to Verify Consumer Preferences for the Developed Product		Use of Expert Tests Performed by a Panel of Trained Experts to Determine the Quality of Products	
Company size (number of employees in organization)		%		%		%
1–10	Yes	52.08%	Yes	54.17%	Yes	33.3%
	No	47.92%	No	45.83%	No	66.7%
11–50	Yes	47.83%	Yes	39.13%	Yes	30.4%
	No	52.17%	No	60.87%	No	69.6%
51–100	Yes	83.33%	Yes	66.67%	Yes	58.3%
	No	16.67%	No	33.33%	No	33.3%
	-	-	-	-	N/A	8.3%
101–1000	Yes	95.24%	Yes	80.95%	Yes	57.1%
	No	4.76%	No	19.05%	No	42.9%
1001–5000	Yes	100.00%	Yes	100.00%	Yes	50.0%
	No	0.00%	No	0.00%	No	50.0%
Above 5000	Yes	83.33%	Yes	91.67%	Yes	58.3%
	No	16.67%	No	8.33%	No	41.7%

## Data Availability

Data is contained within the article.
